# Preventive chemotherapy as a strategy for elimination of neglected tropical parasitic diseases: endgame challenges

**DOI:** 10.1098/rstb.2012.0144

**Published:** 2013-08-05

**Authors:** Moses J. Bockarie, Louise A. Kelly-Hope, Maria Rebollo, David H. Molyneux

**Affiliations:** Centre for Neglected Tropical Diseases, Liverpool School of Tropical Medicine, Pembroke Place, Liverpool L3 5QA, UK

**Keywords:** preventive chemotherapy, neglected tropical diseases, elimination, drug donations

## Abstract

Global efforts to address neglected tropical diseases (NTDs) were stimulated in January 2012 by the London declaration at which 22 partners, including the Bill & Melinda Gates Foundation, World Bank, World Health Organization (WHO) and major pharmaceutical companies committed to sustaining and expanding NTD programmes to eliminate or eradicate 11 NTDs by 2020 to achieve the goals outlined in the recently published WHO road map. Here, we present the current context of preventive chemotherapy for some NTDs, and discuss the problems faced by programmes as they consider the ‘endgame’, such as difficulties of access to populations in post-conflict settings, limited human and financial resources, and the need to expand access to clean water and improved sanitation for schistosomiasis and soil-transmitted helminthiasis. In the case of onchocerciasis and lymphatic filariasis, ivermectin treatment carries a significant risk owing to serious adverse effects in some patients co-infected with the tropical eye worm *Loa loa* filariasis. We discuss the challenges of managing complex partnerships, and maintain advocacy messages for the continued support for elimination of these preventable diseases.

## 1. Introduction

The term preventive chemotherapy (PC) was introduced by the World Health Organization (WHO) [1], to cover the approach of treating populations at risk of human helminth diseases, to prevent transmission or morbidity of those diseases, with drugs either alone or in combination. Delivery is usually undertaken by mass drug distribution campaigns organized by national health services but delivered by communities, through school-based treatments or via the health services themselves. The funding for these drug distribution programmes comes from a variety of sources, including endemic countries, bilateral donors, international organization trust funds and non-governmental development organizations (NGDOs). Increasingly, the strategy of PC is part of an expanded integrated programme to address the neglected tropical diseases (NTDs), including meeting targets for elimination or control [2], as delineated by World Health Assembly (WHA) resolutions (see the electronic supplementary material, table S1).

The progress of programmes based on PC over the last decade has been spectacular in terms of the numbers of people treated (now with over 700 million individual treatments annually), the increase in donations from the pharmaceutical industry, and the recent bilateral commitments to integrated NTD control/elimination programmes [3–5]. However, this progress creates challenges as programmes seek to achieve targets of elimination or reduced prevalence, incidence and morbidity [6]. This will be the focus of this paper.

## 2. Setting the scene: success of drugs for preventive chemotherapy in control and elimination

Collaborative work between pharmaceutical companies contributing drugs and finances, the WHO [2,7], academic institutions, NGDOs and endemic country governments have produced the tools and strategies necessary to achieve the goal of control or elimination of many NTDs, supported by disease-specific partnerships or alliances ([2,7]; www.unitingtocombatntds.org). The donation of ivermectin (as Mectizan) for the control and now elimination of onchocerciasis in 1987 by Merck & Co. Inc. was a landmark in the history of public health [8–10]. Ivermectin kills microfilaria in the skin and inhibits their release by female worms, reducing transmission of *Onchocerca volvulus* by *Simulium* vectors. To interrupt transmission, a minimum of 15 years annual treatment is required, the duration of life of adult worms. Ivermectin has only limited impact on adult *Onchocerca* worms, but relieves pruritus (itching) and delays the progression of ocular morbidity that can lead to irreversible blindness, in addition to reducing transmission. Mobile teams were initially deployed to distribute ivermectin in the Onchocerciasis Control Programme (OCP) in West Africa as a supplement to vector control in 1988. The effectiveness of ivermectin as a microfilaricide led to the creation of the African Programme for Onchocerciasis Control (APOC) in 1995 [11], targeting disease control in 19 endemic African countries not included in the OCP. APOC's success [12] has been built on the development of community-directed treatment with ivermectin, an approach which shifts responsibility of drug delivery from the health system to distributors selected by the community, who collect the drugs from the health service and decide on the time they would distribute drugs to their community. This is a sustainable approach, with successful coverage maintained over periods as long as 20 years [13], a key issue in the context of the endgame. More generally, community-directed intervention (CDI) can be used to deliver other health needs [14], for example distribution of bed nets and access to drugs for the home management of malaria [15].

Various lines of evidence from onchocerciasis control efforts indicate the potential for NTD elimination using PC alone. The elimination of transmission of onchocerciasis has been achieved in some foci in countries in the Americas using ivermectin alone given twice yearly and more recently four times per year to accelerate the process towards the endgame. Initially, six countries had foci, and around 500 000 people were at risk. Treatment twice a year [16] interrupted transmission first in the Santa Rosa focus in Guatemala [17] and then in small foci in Colombia (Lopez de Micay) [18] and Ecuador [19]. Subsequently, in the Escuintla–Guatemala focus (which had higher transmission levels), transmission was arrested after twice yearly treatment from 2002 to 2007 [20]. In most foci in the Americas, treatment has been stopped and post-treatment surveillance is ongoing to detect recrudescence. This success of elimination using ivermectin alone was reproduced in the African setting in Mali and Senegal [21], where after 15–17 years of treatment with ivermectin in annual or six-month intervals, transmission had been effectively eliminated in 126 previously hyperendemic villages, which has been confirmed by entomological data. A study in Nigeria [22], where initial baseline community prevalence was between 23.1 and 84.9 per cent, reported zero prevalence from two foci in Kaduna State (Birnin Gwari and Kauru/Lere) after 15–17 years annual treatment with 75 per cent coverage (still awaiting entomological confirmation).

Successes stemming from the original donation by Merck & Co. Inc. of ivermectin inspired long-term commitments from other major pharmaceutical companies for other NTDs. Novartis donated curative treatments for leprosy, following the demonstration of the efficacy of multidrug therapy (rifampicin, dapsone and clofomazine). GlaxoSmithKline committed to providing albendazole for the elimination of lymphatic filariasis in 1998, and Merck & Co. Inc. expanded their donation of ivermectin for countries co-endemic for onchocerciasis and lymphatic filariasis. This is necessary because the second drug in the package of annual treatment, diethylcarbamazine (DEC), cannot be used in countries where onchocerciasis is endemic given the risk of complications owing to Mazzoti reactions. These are rapid onset, post-treatment reactions caused by the rapid death of microfilaria parasites owing to inflammatory reactions to both parasite antigens and *Wolbachia* [22,23]. The benefit of ivermectin is that it kills microfilaria more slowly and hence the reactions to microfilarial death are not as severe as those with DEC, thus making ivermectin safer and more acceptable, and is an important consideration for community acceptability and sustainability in the context of the endgame.

Another important helminthic parasite disease, schistosomiasis, caused by species of the genus *Schistosoma*, is reportedly endemic in 78 countries worldwide [7,24,25]. Of the estimated 240 million infections globally, over 200 million occur in Africa although there are foci of *Schistosoma mansoni* in Brazil and smaller countries in the Americas, and of *Schistosoma japonicum* and *Schistosoma mekongi* in Asia*.* Nineteen previously endemic countries have interrupted transmission as a public health problem through a combination of drug treatment, improved sanitation and mollusciding [7,25–28]. A cornerstone for the elimination of morbidity due to schistosomiasis is praziquantel, essentially the only medicine commercially available to treat human schistosomiasis [2]. Despite the expansion in coverage of treatment of schistosomiasis with praziquantel from 12 million people in 2006 to 35 million people in 2011 [29] and increased support for control, schistosomiasis remains a major public health problem [7]. The expansion of the treatment with praziquantel continues, and by 2015 there will be sufficient doses to treat 100 million people per year. Examples of successful elimination maintain optimism, for example transmission in Egypt is now restricted to local hotspots, so that *Schistosomiasis haematobium*-induced bladder cancer has been dramatically reduced as a cause of mortality, and prevalences of both *S. haematobium* and *S. mansoni* have been reduced by some 80 per cent with corresponding reductions in intensities of infection. China has made significant progress in reducing morbidity of *S. japonicum* [25]. Large-scale use of PC has likewise decreased schistosomiasis transmission rates as evinced through reduced environmental contamination by eggs discharged by infected people [30], an impact even more evident with urogenital schistosomiasis [31–33]. As a consequence of such patterns, population-based treatment has increasingly been regarded as a public health tool capable of decreasing and interrupting transmission of infection.

## 3. Partnerships and alliances

In January 2012, a group of partners, including the UK Department for International Development (DFID), the United States Agency for International Development (USAID), the Bill & Melinda Gates Foundation, the WHO, the World Bank and major pharmaceutical companies, made commitments to sustain and expand NTD programmes to control or eliminate 10 NTDs by 2020 (www.unitingtocombatntds.org). The diversity of the partners involved in each disease [7,34–36] and the momentum required to integrate interventions against different NTDs poses many challenges, including the coordination and harmonization of drug application procedures, ensuring the reporting of adverse events to comply with regulatory responsibilities and minimization of import taxes and administrative hurdles to getting drugs to endemic countries.

The partners and alliances serve key roles in advocacy and resource mobilization for NTDs. Inevitably, programmes for elimination or control by PC at either global or regional scales have historically proceeded at different rates driven usually by resource availability and country commitment. The timeline ordinarily includes an initial WHA resolution (see the electronic supplementary material, table S1) or regional resolution, for example for onchocerciasis in the Americas, the development of a constituency of partners, the definition of the problem through mapping of the disease burden, establishment of financing options, development of drug management processes, identification of implementing partners (e.g. ministries of health and NGDOs), maintenance of implementation over several years, undertaking evaluation and monitoring, and finally post-intervention surveillance and verification of interruption of transmission or absence of morbidity. These activities are all essential components if the endgame is to be achieved.

## 4. Implementation challenges

Current practices in the management of NTDs have been influenced by the push for integrated control of the diseases amenable to PC, together with the application of alternative intervention strategies to accelerate the interruption of transmission. Additional intervention strategies recommended in the 2012 WHO roadmap for implementation also include vector control, provision of safe water and improved sanitation and hygiene. Amazigo *et al.* [37] identified 11 major challenges following the experiences of the APOC programme (which focused on sustainable delivery of ivermectin as an objective at that time rather than elimination, but are generally applicable):

(1) maintaining timely drug distribution;
(2) integrating distribution into existing primary healthcare services;
(3) strengthening local health infrastructure;
(4) achieving and maintaining optimal coverage;
(5) establishing and up scaling self monitoring by communities;
(6) implementing operational research;
(7) ensuring adequacy of community distributors;
(8) increasing involvement of local NGDOs;
(9) achieving financial sustainability;
(10) implementing equitable cost recovery systems; and
(11) engaging in effective advocacy.

Kyelem *et al.* [38] analysed data from eight countries and identified 40 factors necessary for successful control of lymphatic filariasis. The most important ones were the initial level of *Wuchereria bancrofti* endemicity, the vectorial capacity of the mosquito vector, the mass drug administration (MDA) regimen and the population compliance. The authors identified biological and programmatic factors as the core drivers (table 1). Here, we detail some of the main issues.

**Table 1.** Summary of challenges to elimination and the ‘endgame’ for preventive chemotherapy-targeted diseases.

disease	biological	socio-geographical	logistic	strategic	technical
onchocerciasis	highly efficient vector (*Simulium*) in Africa with long flight range hence risk of reintroduction of infection in *Loa loa* co-endemic areas, serious adverse events (*Loa* encephalopathy) in patients with high *Loa* parasitaemias associated with ivermectin use lack of drug that kills adult worms (macrofilaricide) which can be used in MDA	cross-border issues related to access to treatment for nomadic and refugee populations hard-to-reach communities in post-conflict countries may not have regular access to MDA through community-directed intervention lack of incentives for community drug distributors might reduce coverage	high cost of monitoring and evaluation at low infection levels including post-elimination surveillance	single annual treatment may not be adequate to achieve elimination in many parts of Africa. Possible twice annual treatment with ivermectin to reduce time required for MDA to achieve end game effective selective deployment of doxycycline as macrofilaricide to kill or sterilize adult worms under medical supervision where risk of reduced ivermectin efficacy exists	skin snip increasingly unacceptable due to HIV risk and reduced sensitivity as intensity of infection declines limited laboratory capacity in endemic countries to perform novel diagnostic molecular and serological methods lack of sensitive tools to detect early infection in humans
lymphatic filariasis	persistence of infection in areas where the more efficient culicines are the main vectors serious adverse events associated with ivermectin in *L. Loa* co-endemic areas (see above) lack of macrofilaricides amenable to MDA strategy	persistent non-complaince with MDA by some individuals reduces likelihood of elimination effective coverage in hard-to-reach populations living in extensive groups of islands (e.g. Indonesia), remote hard-to-reach communities (e.g. Papua New Guinea), in post-conflict countries (e.g. DRC) or urban settings (e.g. Dar es Salaam)	high cost of monitoring and evaluation at low infection levels using transmission assessment surveys including post-MDA and elimination surveillance	long-term government commitment lacking resources for scaling up lacking in several large countries with large populations still at risk; and maintaining national coverage for 5–7 years ensuring introduction of integrated vector management to enhance transmission control particularly in *Anopheles* transmission areas use of doxycycline as a macrofilaricide in areas where transmission control via MDA has proved difficult	night blood surveys required as parasites only detectable when they peak in blood between 22.00 and 02.00 h non-competitive market for antigen detection card test (ICT) which detects adult worm antigen; currently provided by single supplier; test does not need cold chain and can be read at 10 min. ICT positivity persists in the absence of infectious agent lack of universally applicable antibody test to detect early infection
schistosomiasis	intermediate host present in water bodies, which populations have to contact as part as their daily activities zoonotic infection in Asia makes elimination difficult significance of various animal reservoirs needs to be assessed (outside China, where water buffalo is dominant reservoir of *S. japonicum*)	poor sanitation facilities prevent behavioural change to prevent faecal and urine contamination of water bodies	current production levels of praziquantel do not meet the demand	implementation of complex strategy involving sectors other than health	rapid diagnosis test to measure *Schistosomiasis haematobium* currently unavailable lack of sensitivity of stool microscopical examination tests following treatment no formulation of praziquantel for pre-school-aged children
soil-transmitted helminths (STH)	eggs that are resistant and remain viable in environment for long periods of time some STHs are not sensitive to available drugs used in PC	poor sanitation facilities prevent behavioural change to prevent faecal and urine contamination of water bodies		implementation of complex strategy involving sectors other than health	lack of sensitivity of parasitological stool tests after treatment

### (a) Mapping pathogen distribution

A principal need of any programme is to define the geographical extent of the problem and define where the public health problem is greatest. This entails mapping each disease to provide estimates of the number requiring treatment and the implementation units and transmission zones. Following mapping, surveys are required to define baseline epidemiological information for sentinel sites, which are essential to follow progress towards the defined epidemiological endpoints post-intervention and following a period of intense surveillance to ensure the endgame scenario has been achieved. The approaches to mapping for the PC-amenable diseases while different for each disease are being integrated when possible, while rapid techniques of assessment of prevalence are available to define where implementation is required [39,40].

A top priority identified for the African region in order to accelerate the progress towards elimination of lymphatic filariasis and onchocerciasis [36] is resolving the treatment challenges posed by the co-endemicity of *Loa loa.* Ivermectin treatment for onchocerciasis or lymphatic filariasis given to people with high parasitaemias of *L. loa* can result in severe adverse events (SAEs) [41–43]. Lymphatic filariasis and *L. loa* co-endemic areas at high risk of SAEs would benefit by PC with albendazole alone (400 mg twice per year) to avoid the use of ivermectin. Where this is the case, the efforts can be enhanced by reducing the vector density [44–46]. Recent maps of the distribution of *L. loa* produced from the Rapid Assessment Procedure for Loiasis [47] and of priority for ivermectin distribution developed from Rapid Epidemiological Mapping for Onchocerciasis help identify areas at high risk of SAEs associated with *Loa* encephalopathy. For more effective deployment of resources, endemic areas may require finer scale mapping (e.g. micro-stratification) [48] to reveal the distribution of various parasites and also current drug distributions patterns [45] to identify where alternative intervention strategies are required, likely to be increasingly crucial as incidence declines. Such approaches will also reveal potential synergies and benefits, for example between the lymphatic filariasis elimination and onchocerciasis control programmes and identify areas where achievement of endgame objectives can be more easily achieved, and conversely where additional approaches are required to reach epidemiological targets.

### (b) Assessing coverage

Since the basis of PC resides in the need to ensure sustained high geographical and therapeutic coverage of the eligible population over a period of years, knowledge of parasite distribution and therapeutic coverage achieved in terms of doses successfully delivered is essential. Geographical coverage is defined as the proportion of the implementation units of infected communities which require treatment for diseases targeted. The calculations of therapeutic coverage in reported figures are based on the total population in any given setting.

MDA programmes dependent on community- or school-based distribution can often play an important role in providing accurate estimates of the true population size for general use by national authorities. However, in post-conflict countries with significant disruption of population structure, extensive migration and resettlement mean estimates of population figures are necessarily open to question. The WHO provides annual reports of the total numbers of people treated for each disease and endemic country [6,7] as well the proportion of those treated compared within the target population. Parker & Allen [49,50] have questioned the validity of this reporting on the basis of studies in Tanzania and Uganda indicating far lower coverage in some districts than is reported, possibly due to inadequate social mobilization and health education. These views were challenged by Molyneux & Malecela [3] based on the fact that the overall epidemiological results show consistent impact of MDA in so many settings. Validation of coverage estimates is desirable, but detailed coverage surveys require an ongoing commitment of resources that are likely to increase during the endgame as incidence, prevalence and intensity of infection fall making monitoring, evaluation and surveillance on a large-scale both technically and financially challenging. The need for culturally appropriate and targeted health education is essential if the endgame is to be reached within the timescales of the WHO roadmap targets.

### (c) Leveraging synergy between different programmes

It has been shown that CDI over 5–6 years with 65 per cent or more coverage could significantly reduce the prevalence and intensity of other filarial parasites, mainly *W. bancrofti* [51,52] and some soil-transmitted helminthiases [53,54]. Similarly, repeated doses of albendazole used in soil-transmitted helminthiasis programmes, particularly for school-aged children, could potentially impact on *W. bancrofti* prevalence. Equally, the scale up of lymphatic filariasis elimination programmes using ivermectin and albendazole across large regions of Africa could help to reinforce the achievements of onchocerciasis control. This would assist in reducing the potential residual human reservoir population of *O. volvulus* and preventing introduction into areas where the disease has been eliminated. In Haiti, the widespread distribution of albendazole within lymphatic filariasis programmes reduced the prevalence and intensity of soil-transmitted helminths, including *Ascaris lumbricoides*, *Trichuris trichiura* and hookworm [53]. However, in contrast to Sri Lanka, Gunawardena *et al.* [55] showed limited and non-significant changes in prevalence in school-aged children examined although the compliance reported was only 59 per cent. In Zanzibar, Mohammed *et al.* [54] examined records from 50 health centres over a 6-year period starting prior to the initiation of MDA for lymphatic filariasis, and recorded that the impact of PC was a dramatic decline in both reported cases and required treatments for worm infections and scabies. Harnessing these types of synergies may be important during the endgame where interest in treatment for infections whose rarity makes them seem negligible wanes.

### (d) Reaching populations

#### (i) Elimination and remote and migrant populations

Despite the progress towards the elimination of onchocerciasis in the Americas, some foci still remain, for example recently discovered newly infected communities in the Amazon. These communities are mobile, making the endgame in such inaccessible environments a serious challenge not just in initiating treatment but also in monitoring and post-treatment surveillance. Of the six countries where onchocerciasis was endemic, transmission remains ongoing in foci in the Amazon rainforest on the border between Venezuela and Brazil where there is limited access, making the indigenous Yanomami populations ones who are most at risk [56]. The major challenge is ensuring high coverage ivermectin treatment four times a year to mobile populations who live in most inaccessible areas. Alternatively, given the goal to stop transmission in the Americas by 2015, the newly discovered infected communities could be treated with doxycylcine which might be the most effective way to achieve the endgame.

### (e) Urban disease elimination

In contrast to onchocerciasis, lymphatic filariasis and schistosomiasis are transmitted in urban settings [57], providing a different set of challenges for organization of drug distribution campaigns. In large cities such as Dar es Salaam, Lagos or Accra, where the population exceeds several million people, reaching a high coverage is both difficult and costly. The major vector of *W. bancrofti* in East African urban settings such as Dar es Salaam is *Culex*, while *Culex* appears not to be susceptible to *W. bancrofti* in West African cities [58]. *Culex* control in urban areas is difficult; hence the achievement of the endgame in Dar es Salaam, for example, will be a challenge as vector control of larval habitats cannot be successfully deployed on sufficient scale. By contrast, *Anopheles gambiae* and *Anopheles funestus* are the dominant rural vectors of *W. bancrofti* [59] whose control will be enhanced by use of impregnated bednets, with the effect of reducing duration of MDA required during the endgame.

Overall, the lymphatic filariasis endgame scenario will be determined by the vectorial capacity of the dominant vector (or in West Africa the lack of susceptibility of *Culex*), the coverage and duration of MDA, the extent of the deployment of insecticide impregnated bednets or long-lasting impregnated nets (LLINs) for malaria control, and, if feasible, the use of a macrofilaricidal drug the most promising being doxycycline [60,61]. In addition, the impact of migration from rural areas to cities in Africa will be a factor in driving the urban prevalence given the extensive prevalence observed in mapping studies in rural areas [62–64]. This presents a dilemma in deciding a treatment strategy if there is no active urban transmission. The complexity of urban environments in terms of transient populations, and areas where there are immigrants from different rural localities, provides challenges in dealing with this heterogeneity in terms of sampling, access and the costs of any evaluation and surveillance activities.

The problems of urban transmission and endgame scenarios are also applicable to schistosomiasis. Recent studies in Cote d'Ivoire, Nigeria and Zambia have all highlighted the challenges of both *S. mansoni* and S. *haematobium* transmission in peri-urban and urban settings [57,65,66]. However, there will be persistent problems of soil-transmitted helminthiasis morbidity in urban and peri-urban areas because of deficient water and sanitation. Therefore, treatment of urban populations of school-aged children with anti-helminthics will be required until the water and sanitation services are sufficiently robust and sustained in parallel with reinforced efforts to change behaviour. A problem in peri-urban settings is the extensive use of human faecal material (night soil) contaminated with helminth eggs as a fertilizer for food and vegetables, thereby maintaining high levels of transmission. *Onchocerca volvulus* transmission does not occur in urban situations.

### (f) Reservoirs

In Asia, schistosomiasis is caused predominantly by *S. japonicum*, a zoonosis. Some 40 mammalian species are hosts although only 10 are considered to contribute to transmission to humans [67]. This situation requires a more integrated approach to control as PC alone cannot lead to elimination. In China, where the principal reservoir hosts are cattle and water buffalo, Wang *et al.* [68] have described the measures used to reduce transmission which include chemotherapy with praziquantel, removal of water buffalo from snail-infected grasslands and their replacement with mechanized farm equipment, improving sanitation and the implementation of an intensive health education programme. In addition to other measures, there is the prospect of vaccinating water buffalo and cattle with an anti-schistosome vaccine [69]. However, additional animal reservoirs—dogs, rodents, sheep and pigs—which cannot be targeted for control make this strategy inapplicable for the other Asian schistosome of humans (*S. mekongi* found in Cambodia and south Lao PDR [27,67]) and in the Philippines. There are no animal reservoirs of onchocerciasis or lymphatic filariasis caused by *W. bancrofti*.

### (g) Vector control and integrated vector management

A key factor that commonly determines outcomes of NTD control is the initial efficiency and abundance of the vector populations. Permanent elimination appears to have been possible in the case of onchocerciasis, where vector populations are isolated. On the island of Bioko, in Equatorial Guinea, the vector, a specific form of *Simulium yahense* [70], was eliminated by helicopter larviciding. In Uganda, the vector *Simulium neavei* in the isolated Itwara focus [71] was controlled by ground larviciding with temephos, starting in 1995. Previously, control efforts had been annual CDI with ivermectin, but after 4 years, transmission was still considerable; the number of infective bites per year was estimated to be 4500–6500, and 40 per cent of parous flies *S. neavei* harboured developing *O. volvulus* larvae. The impact of the vector control in Itwara was monitored by the examination of the crab population for the larvae of *S. neavei*, which have a phoretic association with a particular crab *Potamonautes aloysiisabaudiae.* Transmission appears to have been halted in 2001 and no infested crabs have been found since 2003 by vector control [71].

For more widespread vector populations, significant impacts on multiple NTDs can be achieved via vector control [72], particularly in the context of malaria control programmes. Huge numbers of insecticide-treated nets (ITNs) and LLINs have been distributed as part of malaria control efforts, which has reduced the prevalence of *W. bancrofti* and led to elimination or near elimination of transmission in the Solomon Islands [73]. Similar impacts on other NTDs have also been observed in Nigeria [74], Kenya and Uganda [75,76] and in high transmission areas of Papua New Guinea [77,78]. Bockarie *et al.* [44] and van den Berg *et al.* [45] argue that vector control can supplement MDA and foreshorten the need for MDA given the impact of vector control on transmission parameters such as annual biting rates and annual transmission potentials.

Vector control targeting *Anopheles* is largely dependent on synthetic pyrethroids, the single insecticide recommended for net impregnation and the dominant insecticide class used for indoor residual spraying (IRS). However, resistance to pyrethroids is already widespread in parts of West and Southern Africa [79,80]. To increase insecticide choice for IRS, the organochlorine dichlorodiphenyltrichloroethane (DDT) has been re-introduced, following almost three decades of suspended use [81] due to resistance affecting more than 50 species of *Anopheles* [82]. Alarmingly, DDT resistance has re-emerged in West Africa and was recently detected as far south as Zambia [83]. Resistance may already signify operational significance for the rollout of LLINs for malaria control and is likely to become a serious impediment to the objectives of malaria control or elimination in coming years.

At present, the use of either pyrethroids in bednets or IRS with any single insecticide is effectively a ‘monotherapy’. Evolution is a powerful opponent, and history suggests that insecticide monotherapy is likely to fail, as does chemotherapeutic monotherapy. Unfortunately, few cost-effective alternative insecticides currently exist. LLIN efficacy is thus likely to decline as pyrethroid resistance spreads, potentially approaching the minimal levels of low benefit provided by un-impregnated bednets as shown by studies in Papua New Guinea [78]. Consequently, there is likely to be a limited window of opportunity for the lymphatic filariasis programmes to benefit from ITNs/LLINs [45,84] via integrated vector management, particularly in the context of the endgame.

### (h) Coverage and compliance: social mobilization

The challenge as the endgame approaches is the maintenance of community enthusiasm for delivering drugs. Current problems are that incentives are provided by other programmes and the pressure on ‘volunteers’ to deliver other health interventions [14]. The issue of providing a form of remuneration is an ongoing debate, but one that may impact on the ability of PC interventions to achieve the requisite coverage over the years needed to achieve the endgame scenarios. Training of community workers, social mobilization and information, education and communication (which is often locally specific) are continuing costs to programmes, and require the attribution of adequate resources as a proportion of health budgets. There is also a need to seek to reduce the attrition of community distributors given the investment in their training and the value of their role in the community. Studies clearly demonstrate that should coverage fall below 65 per cent—the estimated level of the total population—it is likely that the MDA annual cycles will need to be extended for several years to achieve the requisite endpoints in both lymphatic filariasis and onchocerciasis [13,85].

Compliance is also important and should be differentiated from coverage. Compliance refers to the frequency with which individuals comply to take the provided drugs, and usually only a proportion of the eligible population are found to have complied with all treatment rounds. It has also been found that there are systematic non-compliers who will thus remain a source of continued infection. Identifying systematic non-compliance and reinforcing the value of compliance within the social mobilization and health education activities at the community level is critical if the criteria for cessation of MDA are to be achieved.

Furthermore, while the current recommendations based on thresholds of prevalence of infection remain valid for achieving control of schistosomiasis-associated morbidity and achieving elimination of schistosomiasis as a public health problem, a more intensified strategy is required in areas where the aim is that of interrupting transmission, which may include more frequent treatment or even focal mollusciciding using ‘niclosamide’ [86]. An integrated approach is required through enhanced efforts to promote behaviour change as well as increased provision of clean water and improved sanitation. The WHO [7] sets out the details of the goals, interventions, targets and the timeline for schistosomiasis elimination towards 2020 while Rollinson *et al.* [25] discuss the background and challenges the elimination agenda presents, in particular the lessons from previous successes and the options that the goal of elimination presents in the many different settings.

### (i) Exclusion criteria

Most drugs used in PC have exclusion criteria, including pregnancy (all drugs), age (children under 2 years for albendazole; less than 90 cm in height for ivermectin as a surrogate for less than 5 years; and less than 94 cm for praziquantel) and the very sick. Clearly, this leaves behind a potential untreated human reservoir for the pathogen, beyond that which simply results from poor coverage (i.e. if treatment is via school, coverage may be low because not all children attend schools).

For schistosomiasis, there is evidence that pre-school-aged children in highly endemic areas are at risk of schistosomiasis infection (a study in Uganda that used parasitological examination of stools and urine dipsticks found prevalences between 32 and 40 per cent [87]), but they are not targeted by MDA. Rather, the WHO currently recommends that children under 4 years of age be treated individually in health facilities as part of the national control programme [88]. This is because there is currently no appropriate formulation of praziquantel to administer to this age group.

### (j) Future drug improvement, drug resistance and the endgame

Ivermectin has been an exceptionally valuable public health product for lymphatic filariasis and onchocerciasis control/elimination allied to albendazole and DEC for lymphatic filariasis, but elimination programmes could be dramatically foreshortened by a drug which kills adult worms. The most exciting prospect lies in the proven macrofilaricidal efficacy and sterilizing effect of the antibiotic doxycycline which targets the obligatory endosymbiont, *Wolbachia*, present in many filarial parasites [61]. If given over a period of four weeks, doxycycline will kill or permanently sterilize adult *Onchocerca* and *Wuchereria* [22,60], but children under 9 years old and pregnant women are ineligible for treatment, and hence are a potential reservoir. Community studies demonstrate not only efficacy but a high level of compliance to programmes involving this treatment [89]. There are ongoing studies to reduce the duration of treatment and search for alternative antibiotics that target the symbiont *Wolbachia* [61], as this approach to elimination holds promise where the community-directed approach with ivermectin has been less effective and where there may be a potential loss of efficacy or true resistance [90]. Doxycycline or similar anti-*Wolbachia* antibiotics provide tools of considerable value in achieving the endgame of killing or sterilizing adult worms and the epidemiological endpoint for both lymphatic filariasis and onchocerciasis. To address the endgame challenges among onchocerciasis foci in Venezuela and Brazil, macrofilaricidal treatment (test and treat with a six-week course of doxycycline 100 mg per day, which kills adult parasites) is being considered to overcome the limitations of ivermectin.

## 5. Resources: financial, human capacity and country commitment

A constraint in any health programme is that only finite resources are available. The NTDs are no exception, and programmes are constrained by the lack of available resources despite availability of the donated drugs. Recently, the USAID and the UK DFID significantly increased their commitments to the control and elimination of these diseases as stated in the London Declaration (www.unitingtocombatntds.org). The Bill & Melinda Gates Foundation committed US$ 340 million over the coming 5 years to support research into NTDs. NGDOs continue to raise finance for implementation of country programmes in onchocerciasis, lymphatic filariasis and trachoma. Increasing recognition by endemic countries is needed to incorporate NTD control into national health plans, budgets and poverty reduction strategies to ensure sustainable finance. The APOC programme continues to enjoy support from a consortium of donors and has been able to support NTD programmes in countries endemic for onchocerciasis since 2006. The annual APOC budget of around US$ 20 million is administered through a World Bank Trust Fund, and bilateral donors have committed to support the new objective of elimination of onchocerciasis financially until 2015. The bilateral support from the USAID is directed to ensuring that integrated NTD programmes are initiated in selected endemic countries, with countries themselves contributing a proportion of the costs of programmes through provision for example of staff, office and laboratory facilities.

A particular need in NTDs and a challenge for the expansion of future programmes is the availability of human resource capacity. Throughout the domain of global health, the lack of human resources at all levels of the health systems in poorer countries is recognized as a barrier to a better functioning of health services. Notwithstanding potential technical (reduced efficacy of drugs), political (conflict or civil unrest) and environmental (global climate impacts) challenges, without the requisite country skills from the central level to the community, no programme can effectively function and this needs to be addressed as a priority. The process of identifying the barriers to enhanced capacity strengthening requires a situation analysis with stakeholders to provide a basis for future planning to deliver the human resources to deliver the endgame of elimination. Two essential issues emerge: research capacity [91,92] and implementation capacity [7]. The need for implementation capacity will drive the achievement of the WHO roadmap targets. However, the research training is required within endemic as well as developed countries' academia and research institutions that focus on the search for new tools. However, the priority must be to enhance the capacity needed to implement the existing tools to achieve further scale up of PC and associated morbidity control (e.g. surgery for trachoma and lymphatic filariasis). There must also be the recognition that timelines to deployment of tools that will contribute effectively to achieving the endgame should be introduced into practice as soon as safe and practicable. Too often there are significant delays in translating research findings into practice. Such delays can postpone the achievement of the endgame for several diseases.

## 6. Conclusions

We have identified the challenges to the implementation of PC programmes to control or eliminate diseases responsible for a significant health burden in some of the world's poorest countries. PC is based on efficacious drugs, the majority of which are donated and provided on a long-term basis by several major pharmaceutical companies—some ‘as long as needed’. A significant increase in the number of treatments has been achieved over recent years with over 700 million people receiving the necessary drugs each year [7]. However, to achieve the goals set by WHA resolutions (see the electronic supplementary material, table S1) and the endpoints identified in the WHO roadmap, the further commitment of resources will be required to achieve the target annual treatment of at least one billion for the coming 5–10 years [7].

The increasing use of donated drugs when dealing with a single strategy of control or elimination for diseases in different regional settings creates significant complexity both technically and from a policy perspective. To ensure that endemic countries have a central role in all aspects of these programmes, there should be coordination and communication among the partnership involved in financing, implementation, research and health policy.

The challenges of partnership, resourcing and communication are not unique to NTDs nor are the particular challenges of up scaling to ensure the necessary geographical coverage and timely implementation of annual distribution. Given that the drugs used in these programmes have synergistic impacts, there becomes an increasing complexity in analysing the impact of programmes. Evaluation and monitoring to ensure post-control surveillance is critical, and will require additional financing.

There must be a continuous emphasis on improving the efficiency of the PC strategy, by developing improved monitoring and surveillance tools, seeking alternative drugs in the event of loss of efficacy or resistance, ensuring effective reporting systems and maintaining as high a coverage as possible. Approaches to ensuring sustained high coverage often over many years will need health education programmes to fit local social and cultural settings. At the global policy level, the challenge of ensuring that continued bilateral funding is available, through an emphasis on the key advocacy messages about the impact of these diseases as drivers of poverty and our ability through highly cost-effective interventions to reduce the burden or even eliminate them, is critical. High profile advocates will remain an important means of emphasizing that investment in NTD control and elimination is a major contribution to poverty alleviation.
